# Establishment of HSV1 Latency in Immunodeficient Mice Facilitates Efficient *In Vivo* Reactivation

**DOI:** 10.1371/journal.ppat.1004730

**Published:** 2015-03-11

**Authors:** Chandran Ramakrishna, Adrianna Ferraioli, Aleth Calle, Thanh K. Nguyen, Harry Openshaw, Patric S. Lundberg, Patrick Lomonte, Edouard M. Cantin

**Affiliations:** 1 Department of Virology, Beckman Research Institute of City of Hope; Duarte, California, United States of America; 2 Department of Microbiology and Molecular Cell Biology, Eastern Virginia Medical School, Norfolk, Virginia, United States of America; 3 Centre de Génétique et Physiologie Moléculaire et Cellulaire CNRS UMR5534, Université de Lyon 1, Lyon, France; 4 Université de Lyon 1, Lyon, France; 5 Laboratoire d’excellence, LabEX DEVweCAN, Lyon, France; 6 Department of Neurology, Beckman Research Institute of City of Hope; Duarte, California, United States of America; 7 Department of Immunology, Beckman Research Institute of City of Hope; Duarte, California, United States of America; University of Glasgow, UNITED KINGDOM

## Abstract

The establishment of latent infections in sensory neurons is a remarkably effective immune evasion strategy that accounts for the widespread dissemination of life long Herpes Simplex Virus type 1 (HSV1) infections in humans. Periodic reactivation of latent virus results in asymptomatic shedding and transmission of HSV1 or recurrent disease that is usually mild but can be severe. An in-depth understanding of the mechanisms regulating the maintenance of latency and reactivation are essential for developing new approaches to block reactivation. However, the lack of a reliable mouse model that supports efficient *in vivo* reactivation (IVR) resulting in production of infectious HSV1 and/or disease has hampered progress. Since HSV1 reactivation is enhanced in immunosuppressed hosts, we exploited the antiviral and immunomodulatory activities of IVIG (intravenous immunoglobulins) to promote survival of latently infected immunodeficient Rag mice. Latently infected Rag mice derived by high dose (HD), but not low dose (LD), HSV1 inoculation exhibited spontaneous reactivation. Following hyperthermia stress (HS), the majority of HD inoculated mice developed HSV1 encephalitis (HSE) rapidly and synchronously, whereas for LD inoculated mice reactivated HSV1 persisted only transiently in trigeminal ganglia (Tg). T cells, but not B cells, were required to suppress spontaneous reactivation in HD inoculated latently infected mice. Transfer of HSV1 memory but not OVA specific or naïve T cells prior to HS blocked IVR, revealing the utility of this powerful Rag latency model for studying immune mechanisms involved in control of reactivation. Crossing Rag mice to various knockout strains and infecting them with wild type or mutant HSV1 strains is expected to provide novel insights into the role of specific cellular and viral genes in reactivation, thereby facilitating identification of new targets with the potential to block reactivation.

## Author Summary

Although mouse models have been very useful in studies of HSV1 latency, the inability to efficiently reactivate latent HSV1 *in vivo* has impeded studies of reactivation. Reasoning that reactivation would be much more efficient in the absence of T cells, we exploited IVIG to promote survival of latently infected Rag mice lacking B and T cells. We established a threshold inoculum dose that was higher for B6- compared to 129-Rag mice, which determined whether HSV1 could be efficiently reactivated *in vivo* resulting in encephalitis. We showed directly that memory T cells are required to control spontaneous and induced reactivation in mice inoculated at high dose but are dispensable for maintaining latency in low dose inoculated mice. Incorporating different knockout strains into the Rag latency model by adoptive transfer of cells or crossbreeding will facilitate studying the role of various cellular genes involved in regulating neuronal gene expression and innate and adaptive immunity in the control of HSV1 reactivation. The potential of this powerful latency model to unravel the molecular and immune mechanisms regulating latency will be realized only after it is adopted and refined by researchers in the field.

## Introduction

Herpes simplex virus type 1 and 2 (HSV1 and HSV2) have colonized roughly 90% and 45% the of US population respectively and are thus important constituents of the human virome. After breaching mucosal defenses, HSV1 invades sensory neurons and travels via axonal pathways to sensory ganglia and eventually to the CNS where lifelong latent infections are established in PNS and CNS neurons [1,2]. During latency, expression of lytic cycle genes are significantly repressed except for abundant expression of the latency associated transcripts (LATs) [2]. In humans frequent, but often asymptomatic, reactivation events result in virus shedding in bodily fluids, which promotes further dissemination of infection in the population. Reactivated HSV1 is the cause of much human suffering and several diseases including most frequently recurrent oral infections and eye infections, that are a major cause of blindness in the USA. HSV1 encephalitis (HSE), though rare, is associated with high mortality (>20%) and devastating neurological consequences in survivors, particularly newborns, despite antiviral treatment [3–5]. A recent epidemiological study suggests that HSV1 has now replaced HSV2 as the most common cause of genital infections [6]. Though less likely to recur, genital HSV-1 is a significant cause of serious neonatal infections including encephalitis, which thus may well increase in the future.

Chronic recurrent orolabial and genital HSV infections occur in some patients resulting in physical disabilities, social isolation and significant emotional trauma [7,8]. Since HSV1 reactivates at higher frequencies in immunocompromised individuals, transplant patients are increasingly being treated prophylactically with acyclovir (ACV) to prevent serious disease. This practice has increased the incidence of ACV resistant (ACVr) strains in patients undergoing long-term immunosuppressive treatment with frequencies exceeding 25% being reported for allogeneic hematopoietic stem cell transplant patients [9–11]. Although long-term ACV suppressive therapy for mucocutaneous disease in immunocompetent individuals has not been associated with emergence of ACVr strains, long-term ACV prophylaxis for recurrent herpetic keratitis (HK) was recently found to be an important risk factor for development of ACVr stromal HK, which could lead to treatment refractory disease and poor vision outcomes for affected patients [12,13]. These considerations emphasize the urgency to understand the regulation of latency at the cellular and molecular levels to accelerate development of new approaches to prevent or reduce reactivation.

A major obstacle to studying reactivation is the lack of a reliable latency model that supports efficient IVR with measureable infectious virus production in sensory ganglia and development of disease. While HS can induce reactivation in susceptible wild type (wt) mice (129, Swiss Webster), major limitations are transient production of very low levels of virus due to rapid mobilization of Tg resident HSV1 specific T cells that suppress reactivation [14–17]. In contrast, latently infected resistant B6 mice are relatively refractory to *in vivo* induced reactivation compared to BALB/c mice [18–20]. As HSV tends to reactivate preferentially in immunosuppressed individuals and experimental animals [20,21], we set out to develop a model of HSV latency in immunodeficient Rag mice that lack B and T cells. Our prior studies, documenting establishment of latency in immunodeficient B6 mice that survived acute infection, supported the feasibility of establishing the model [22].

Intravenous immunoglobulins (IVIG) are a preparation of human polyclonal IgGs obtained from pooled plasma of large numbers of healthy donors. Although initially used as replacement therapy for primary and secondary immune deficiencies, IVIG is now widely used for treating a variety of autoimmune and inflammatory diseases and also viral infections; IVIG’s benefit in these conditions derives from its potent immunomodulatory and antiviral activities [23–26]. We exploited the potent immunomodulatory and antiviral activities of IVIG, which we reported are highly effective in preventing fatal HSE [27], to promote survival of latently infected immunodeficient Rag mice that lack B and T cells. Importantly, IVR of HSV1 in latently infected Rag mice established with high dose (HD) inocula resulted in high rates of fatal HSE. In contrast, reactivated HSV1 persisted only transiently in Tgs of low dose (LD) inoculated mice presumably due to the action of intrinsic neuronal restriction mechanisms [22,28]. The prospect that studies using this innovative latency model will not only reveal the immunologic and molecular mechanisms regulating HSV1 latency but also viral and cellular genes with the potential for development as novel therapeutic targets for controlling reactivation is high.

## Results

Maladapted inflammatory responses to HSV1 infection in the central nervous system (CNS) of susceptible wt 129 mice are the primary cause of fatal HSE [22,29]. A single dose of IVIG given intraperitoneally (ip) 24 h post infection (pi) protected all 129 wt mice inoculated at 10x LD_50_ (3200 PFU) by exerting potent antiviral and immunomodulatory effects that suppressed viral replication and deleterious inflammatory responses [27]. Immunodeficient Rag mice are highly susceptible to HSV1, as they cannot eradicate infectious virus. Our goal was to exploit IVIG to promote survival of latently infected Rag mice because we anticipated that IVR would be significantly enhanced in Rag mice, making this an ideal model for elucidating the molecular and immune mechanisms involved in control of HSV1 reactivation.

### IVIG Protection of Rag Mice Depends on the Inoculum Dose

**Fig 1:**
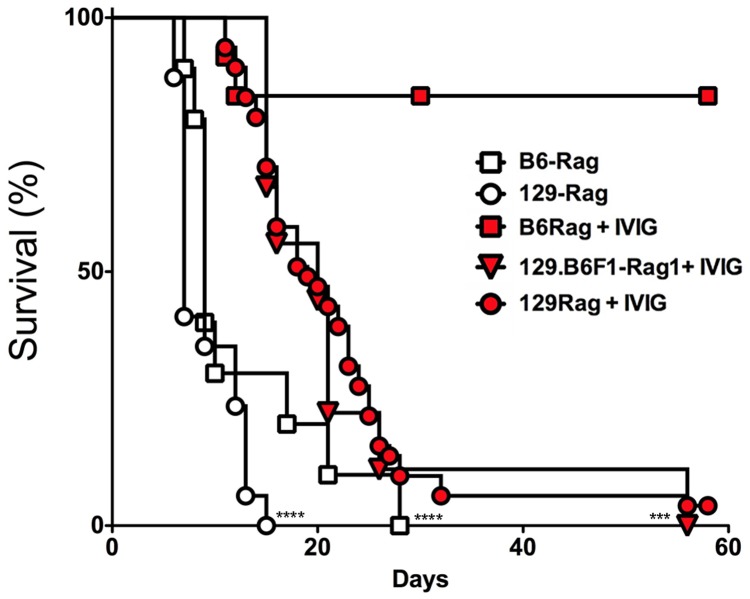
IVIG protects B6 but not 129-Rag^-/-^ mice from HSE. 129-Rag, B6-Rag, and 129.B6F1-Rag mice infected with 3200 PFU HSV1 were given 4 mg IVIG (red) or PBS (open) by ip injections at 24 h pi and observed for survival (2–6 experiments, n = 10–51 mice). The following data sets were statistically significant: B6-Rag+IVIG vs B6-Rag and B6-Rag+IVIG vs 129.B6F1-Rag: ***p = 0.0003; 129-Rag vs 129-Rag+IVIG: ****p<0.0001.

To determine IVIG protection of Rag mice, B6-Rag and 129-Rag mice inoculated with 3200 PFU HSV1 (corresponding to 10x LD_50_ for 129 wt mice) were given a single dose of IVIG 24 h pi and monitored for survival. The majority of B6-Rag mice survived long-term while the 129-Rag mice succumbed, though their survival was prolonged compared to PBS-controls (Fig. 1). Interestingly, cumulative survival for infected B6.129F1 Rag mice was similar to 129-Rag mice but significantly lower than for B6-Rag mice (Fig. 1), which indicates that responsiveness to IVIG is genetically determined.

**Fig 2:**
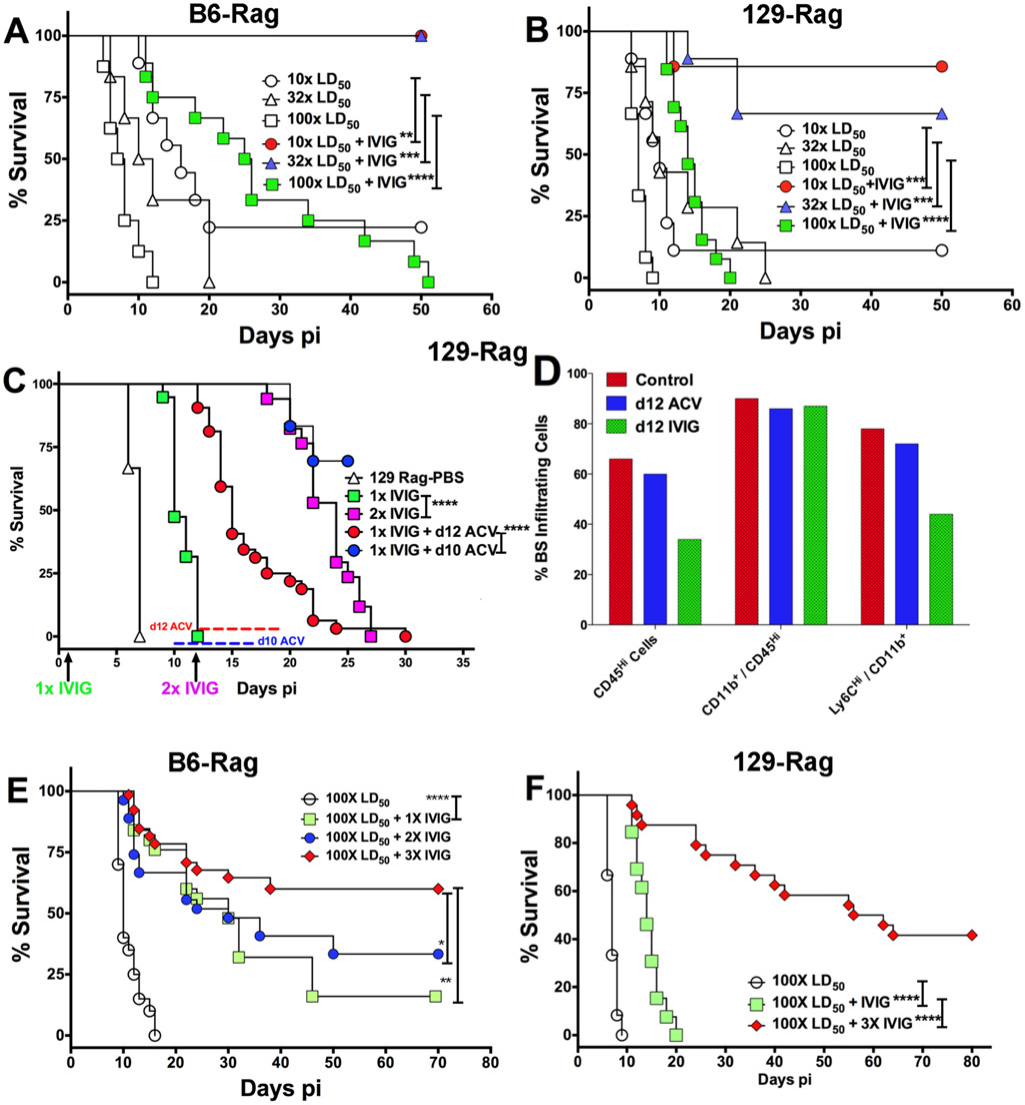
IVIG protection of Rag mice is HSV1 dose dependent. **(A)** B6-Rag or **(B)** 129-Rag mice infected with HSV1 at 10x, 32x or 100x LD_50_ were treated with either PBS or 4 mg IVIG at 24 h pi and monitored for survival (n = 12–28 mice / treatment group). **(C)** 129-Rag mice infected with HSV1 at 100x LD_50_ were treated with PBS or IVIG (1x) at 24 h pi and given either a second dose of IVIG at day 12 pi (2x), or a 7-day course of ACV treatment beginning day 10 pi (blue line) or day 12 pi (red line); (2–4 experiments, n = 12–32 mice / group). **(D)** Mononuclear cells isolated from pooled BS of 4 mice treated with 1x IVIG (control) at day 12 pi, 1x IVIG + d12 ACV (d12 ACV) or 2x IVIG (d12 IVIG) at day 15 pi were analyzed for infiltrating cell subsets by flow cytometry. **(E)** HD infected B6-Rag or **(F)** 129-Rag mice were treated with IVIG at 24 h pi. After the first dose, some mice received 1 (1x), 2 (2x) or 3 (3x) additional doses of IVIG given every 12 days (n = 20–65 mice / group). (*p<0.05 **p<0.01, ***p<0.001, ****p<0.0001).

To account for differential IVIG protection of 129-Rag and B6-Rag mice, we examined the effect of varying the HSV1 inoculum dose. Having determined an LD_50_ of 30 and 100 PFU for 129-Rag and B6-Rag mice respectively, we treated Rag mice infected at 10x, 32x and 100x LD_50_ with PBS or IVIG at 24 h pi and monitored them for survival. A single dose of IVIG protected all B6-Rag mice inoculated at 10x and 32x LD_50_ but not 100x LD_50_ (Fig. 2A). Although, the majority of IVIG treated 129-Rag mice inoculated at 10x and 32x LD_50_ survived, protection was less robust than for B6-Rag mice as shown by the progressive decline in protection with inocula of 32x and 100x LD_50_ (Fig. 2B). All B6-Rag and 129-Rag mice inoculated at 100x LD_50_ succumbed despite treatment with IVIG (Fig. 2A and 2B). Virtually all B6-Rag and 129-Rag mice that survived HSV1 challenge of 10x and 32x LD_50_ (>90%) harbored latent infections in the Tg as revealed by reactivation of HSV1 in Tg explant cultures.

To investigate the effects of IVIG in Rag mice inoculated at 100x LD_50_, mice were sacrificed at different times after infection and viral titers and inflammatory cell infiltrates were determined in the Tg and brainstem (BS) respectively. Virus replication and inflammation were suppressed as early as day 5 pi. By day 8 pi HSV1 was cleared from Tgs of IVIG treated 129-Rag mice, while extensive replication and inflammation were still evident in Tgs and BS of control mice, respectively (Table 1). By day 14 pi, HSV1 replication had resumed in the Tgs of IVIG treated 129-Rag mice in parallel with increased BS inflammation comprised predominantly of inflammatory monocytes, whereas all control mice were dead (Table 1).

**Table 1:** Tg virus titers and BS inflammation in Rag mice.

Days PI	Tg Virus titers^**b**^	BS Inflammation^**c**^		Tg Virus titers^**b**^	BS Inflammation^**c**^	
		CD45^hi^	CD11b^+^		CD45^hi^	CD11b^+^
	**129 Rag mice + PBS** ^a^			**129 Rag mice + IVIG** ^a^		
Day 5	4.0 ± 0.6	42	88	1.5 ± 0.4	20	65
Day 8	3.8 ± 0.3	60	92	ND*	42	72
Day 14	NA**	NA**	NA**	1.2 ± 0.5	65	80
	**B6 Rag mice + PBS** ^a^			**B6 Rag mice + IVIG** ^a^		
Day 5	2.74 ± 0.48	32	95	ND*	10	94
Day 8	2.72 ± 0.16	55	90	0.6± 0.65	22	88
Day 14	NA**	NA**	NA**	0.96 ± 0.89	40	98

^a^ 129- and B6-Rag mice were infected with 3200 or 10^4^ PFU HSV 17+ strain (HD) respectively and treated with either PBS or IVIG at 24 h PI.

^b^Virus titers in Tg depicted as log_10_ PFU.

^c^CD45^hi^ infiltrates depicted as % of total mononuclear cells isolated from the BS; CD11b^+^ cells depicted as % of total BS CD45^hi^ infiltrating cells.

*ND: Not Detectable,

**NA: PBS treated mice were dead at this time point.

To determine if mortality correlated more with CNS inflammation or viral cytopathology, 129-Rag mice infected at 100x LD_50_ then given a single dose of IVIG at 24 h pi were separated into two groups: one group received a 10-day course of daily ACV injections beginning day 12 pi and the other, a second dose of IVIG on day 12 pi. ACV treatment failed to prevent mortality as mice began dying on day 14 pi (Fig. 2C). Though infectious HSV1 could not be detected in the BS or Tg of ACV treated mice, the majority of cells infiltrating the BS of moribund mice on day 15 pi were highly inflammatory Ly6C^hi^ monocytes (Fig. 2D) that we previously reported were causally involved in HSE [27]. In contrast, a second dose of IVIG prolonged survival significantly with the majority of mice dying after day 20 pi (Fig. 2C). Infectious virus was present in BS of the dying mice but notably, pathogenic inflammatory Ly6C^hi^ monocytes were significantly reduced compared to control and ACV treated mice at day 15 pi (Fig. 2D). Thus, IVIG prolonged survival of infected 129-Rag mice by suppressing both HSV1 replication and inflammation in the CNS, while ACV was ineffectual because it inhibited only HSV1 replication. This interpretation is supported by observing a similar prolongation of survival similar to day 12 IVIG when ACV treatment was started two days earlier, which by blocking resumption of HSV replication effectively prevented resurgence of lethal CNS inflammatory responses that occurred when ACV treatment was started on day 12 pi (Fig. 2C and 2D).

IVIG treatment of B6-Rag mice inoculated at 32x and 100x LD_50_ resulted in a similar trend of reduced virus replication and inflammation early after infection, but overall the extent of CNS inflammation was lower than for IVIG treated 129-Rag mice (Table 1 and S1 Fig). Hence for B6-Rag mice compared to 129-Rag mice, IVIG protection was more robust as it depended primarily on suppression of virus replication, whereas suppression of CNS inflammation was more important for 129-Rag mice. For both Rag strains, 32x LD_50_ defines an HSV1 inoculum threshold dose, referred to as low dose or LD. A single dose of IVIG given 24 h pi protected all Rag mice inoculated at or below LD (Fig. 2A and 2B), whereas progressively increased mortality occurred above the LD inoculum, which was 3-fold higher for B6-Rag compared to 129-Rag mice.

### Cyclic IVIG Treatment Promotes Survival of Rag Mice Inoculated at 100x LD_50_

**Fig 3:**
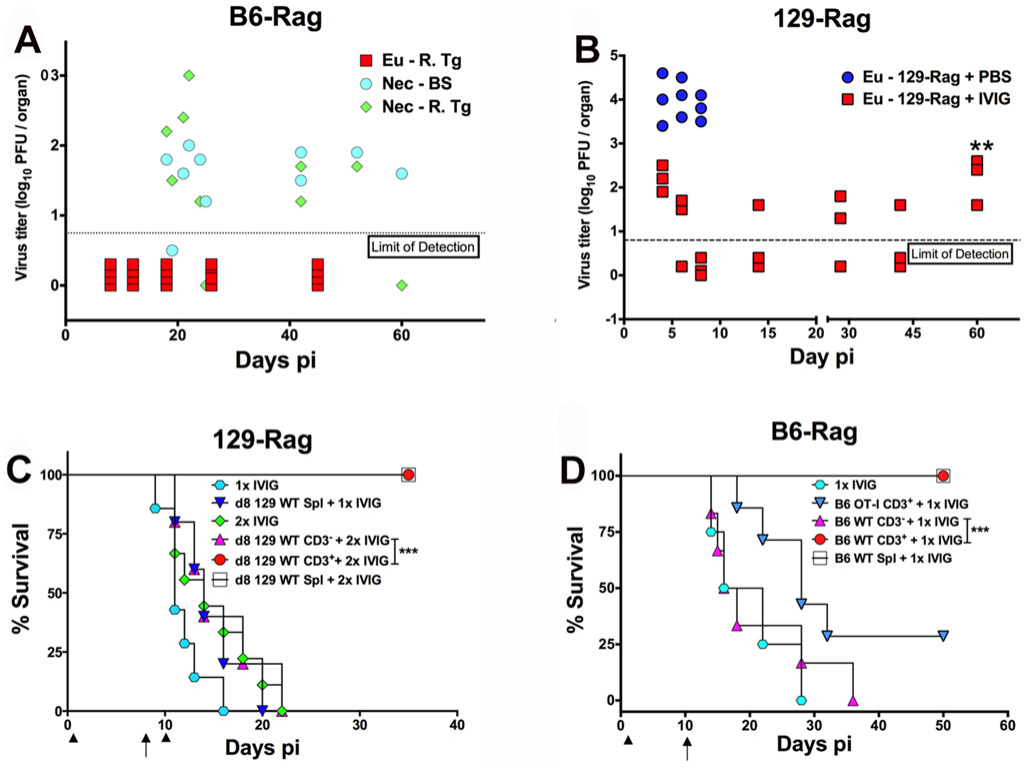
IVIG protection against HD HSV1 infection is T cell dependent. **(A)** HD infected B6-Rag or **(B)** 129-Rag mice treated with 4 cyclical doses of IVIG were euthanized (Eu) at different times pi for determination of virus titers in the right Tg (B6-Rag+IVIG: red squares, Eu-R.Tg; 129-Rag+IVIG: red squares). B6-Rag mice that died were assayed for infectious virus in the right Tg (Nec-R.Tg: green diamonds) and BS (Nec-BS: cyan circles). Virus titers in Tgs isolated from HD infected PBS treated 129-Rag mice (blue circles) are also included in **B**. At day 60 pi (**), the right Tgs from surviving IVIG treated 129-Rag mice were explanted for 5 days and assayed for infectious virus **(B)**; (n = 3–4 mice/ group per time point). HD infected 129-Rag **(C)** or B6-Rag **(D)** mice treated with IVIG at 24 h pi were given splenocytes, purified CD3^+^ or CD3^-^ cell fractions (1–2x10^7^ cells) isolated from naïve 129 or B6 wt or B6 OT-I spleens on day 8 or 10 pi; 129-Rag controls treated 1x or 2x with IVIG or B6-Rag controls treated 1x with IVIG did not receive cells. All 129-Rag recipients received a second IVIG dose on day 10 pi except for one group that received only 1x IVIG (d8 129 WT Spl + 1x IVIG) (n = 6–8 for controls and 10–14 for recipients / group). Arrows = day of cell transfer, arrowheads = day of IVIG injection. ***p = 0.0007; ***p = 0.0005.

Since IVIG induced prolonged survival that could be significantly extended by a second IVIG treatment as shown in Fig. 2C and Table 1, we tested cyclic IVIG treatment every 12 days after the initial dose at 24 h pi as an approach to promote long term survival of latently infected Rag mice inoculated at 100x LD_50_, referred to as high dose or HD mice. Three such cycles of IVIG after the initial dose at 24 h pi resulted in a population of stable latently infected B6-Rag mice (Fig. 2E), as determined by the absence of infectious HSV1 in Tgs and BS of randomly selected mice during the 36 day IVIG treatment interval and a >30 day follow-up period (Fig. 3A). Similarly, roughly 40% of 129-Rag mice treated with three IVIG cycles after the initial dose at 24 h pi survived for >40 days after the last IVIG cycle at day 36 pi (Fig. 2F). However, in contrast to B6-Rag mice, infectious HSV was detected in the Tgs of some mice sacrificed randomly at intervals during and after the last IVIG cycle (Fig. 3B). The occasional deaths that occurred randomly during and after cyclic IVIG treatment were attributed to spontaneous reactivation, as infectious HSV1 was present in the Tg and/or BS of both B6-Rag and 129-Rag mice that died (Fig. 3A). It is important to emphasize that healthy-appearing B6-Rag mice without signs of encephalitis that were randomly sacrificed had no detectable infectious virus in either the Tg or BS (Fig. 3A).

### T Cells Suppress HSV1 and Promote Survival of Rag Mice Infected at HD

We evaluated the capacity of T cells to eradicate replicating HSV1 and thereby promote latency and survival of Rag mice inoculated at HD. We transferred total spleen cells or the CD3^+^ or CD3^-^ fractions from naïve wt mice into HD B6-Rag mice at day 10 pi; 129-Rag mice received transfers at day 8 pi, which is before virus replication resumes in mice given one dose of IVIG 24 h pi (Fig. 2C and Table 1). It was necessary to treat 129-Rag, but not B6-Rag, recipient mice with IVIG because wt 129 mice are highly susceptible to HSE [22] unless treated with IVIG [27]. All infected HD 129-Rag mice infused with 129 wt spleen cells or the CD3^+^ fraction that were then treated with IVIG were protected, while all mice given spleen cells or IVIG alone succumbed (Fig. 3C). All HD inoculated B6-Rag recipients of wt spleen cells, or the CD3^+^ but not the CD3^-^ fraction, survived (Fig. 3D). Antigen specific T cells are required for protection because B6 OT-I transgenic CD8^+^ T cells that only recognize OVA_247–256_ failed to protect IVIG treated HD B6-Rag recipients (Fig. 3D). The slight but insignificant increase in survival of B6-Rag mice transferred with CD3^+^ OT-I cells relative to recipients of wt CD3^-^ spleen cells can be ascribed to other HSV1 specific T cells, including CD4^+^ and γδ^+^ T cells because all HSV1 infected B6 wt and OT-I mice survived.

### High Rates of Fatal HSE after IVR of HSV1 in HD Inoculated Rag Mice

**Fig 4:**
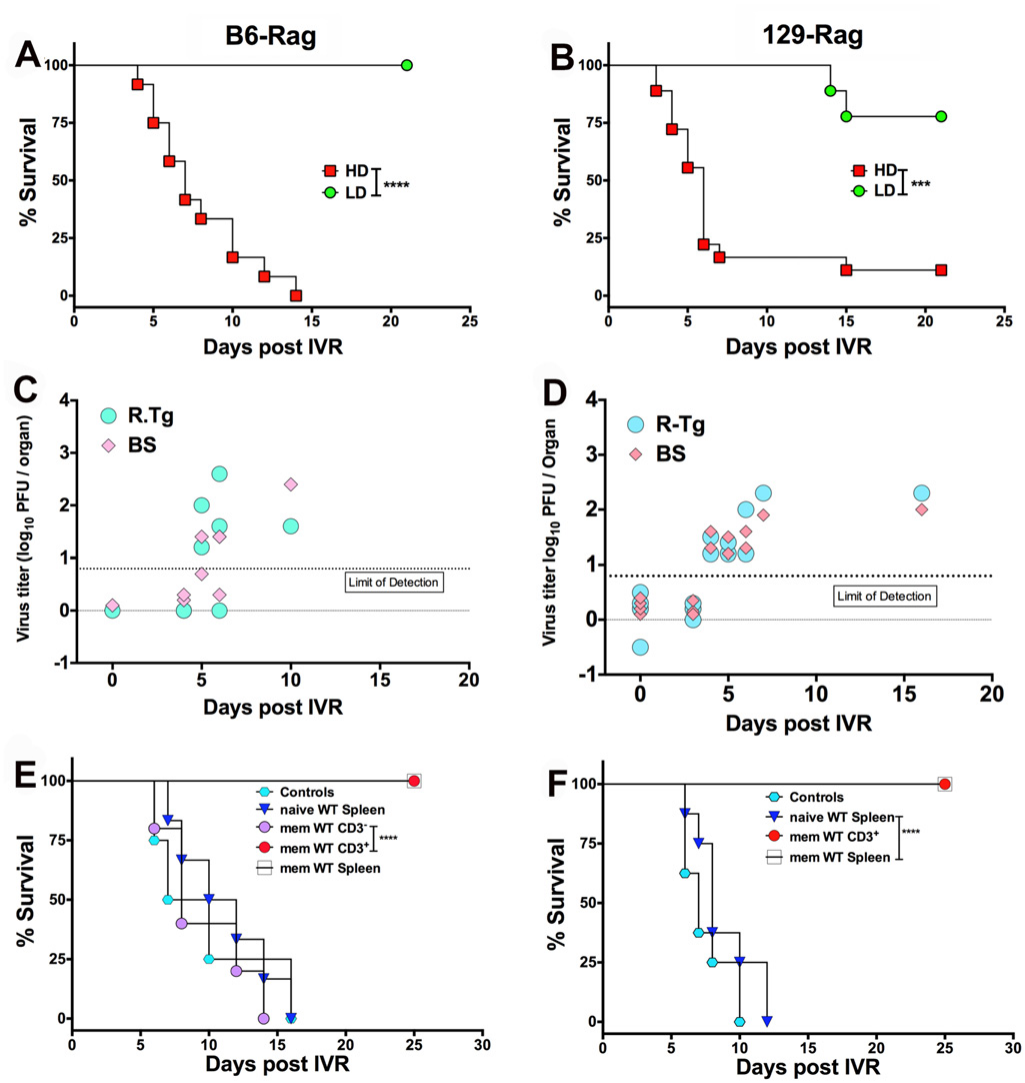
HS induced HSV1 reactivation in HD mice is controlled by T cells. Healthy LD and HD infected B6-Rag **(A)** and 129-Rag mice **(B)** at day 60 pi were subjected to HS to induce IVR and monitored for survival (data represents 2 experiments; n = 8–20 mice / group). HSV1 titers were determined in the right Tg and BS of dying B6-Rag **(C)** and 129-Rag mice **(D)**; base line titers were determined in Tg and BS of 4 control (non-HS treated) mice at day 60 pi (d0 for IVR). **(E)** B6-Rag and **(F)** 129-Rag mice were transferred with 1–2x10^7^ naïve or memory (mem) splenocytes or CD3^+^ T cells isolated from mock or HSV1 immunized B6 or 129 wt mice respectively, 3 days prior to HS and monitored for survival. Data represents 1–2 experiments (n = 8–15 / mice per group). ***p = 0.004, ****p< 0.0001.

We subjected healthy latently infected LD and HD inoculated Rag mice at day 60 pi to HS, which Sawtell [15] reported, and we confirmed [14], induces IVR resulting in transient production of infectious HSV1 detectable maximally 24 h later in Tgs of wt mice. The rapid and synchronous onset of typical signs of HSE (hunched back, ruffled fur, impaired balance and mobility) in HD but not LD Rag mice signified highly efficient IVR that culminated in very high rates of mortality (typically >90%) for both B6-Rag and 129-Rag mice (Fig. 4A and 4B) with high levels of infectious HSV1 in the Tg and/or BS of mice that succumbed (Fig. 4C and 4D). It is important to stress that only healthy mice were selected for IVR and that prior to HS infectious HSV1 was absent from Tgs and BS of healthy latently infected mice analyzed at day 60 pi (Fig. 4C and 4D, day 0 with respect to IVR). Although, LD inoculated B6-Rag or 129-Rag mice subjected to HS did not succumb to HSE, low levels of infectious HSV1 was detected in all Tgs of 129-Rag and B6-Rag mice assayed at 24 h but not 21 days after HS (not shown). Since BS were not assayed we cannot exclude that latent HSV1 was reactivated in the CNS also. Indeed, the recent observation that reactivated HSV1 was first detected in BS rather Tgs of wt B6 mice after IVR makes this a virtual certainty [30]. Notably, infectious HSV1 was not detected in Tgs from LD mice not subjected to HS but virus was detected in all Tgs induced to reactivate in *in vitro* explant cultures.

We determined the latent HSV1 DNA genomic loads by quantitative Taqman PCR analysis for HSV1 gG sequences in Tgs from Rag mice inoculated at LD and HD. For 129-Rag mice there was no difference, but LD B6-Rag mice had a slightly higher load than HD mice (S2 Fig). Thus, differences in the latent HSV1 genomic load in Tgs of LD and HD Rag mice cannot account for the observed differences in development of HSE after HS to LD and HD Rag mice.

### HSV1 Gene Expression in LD and HD B6-Rag Mice

**Fig 5:**
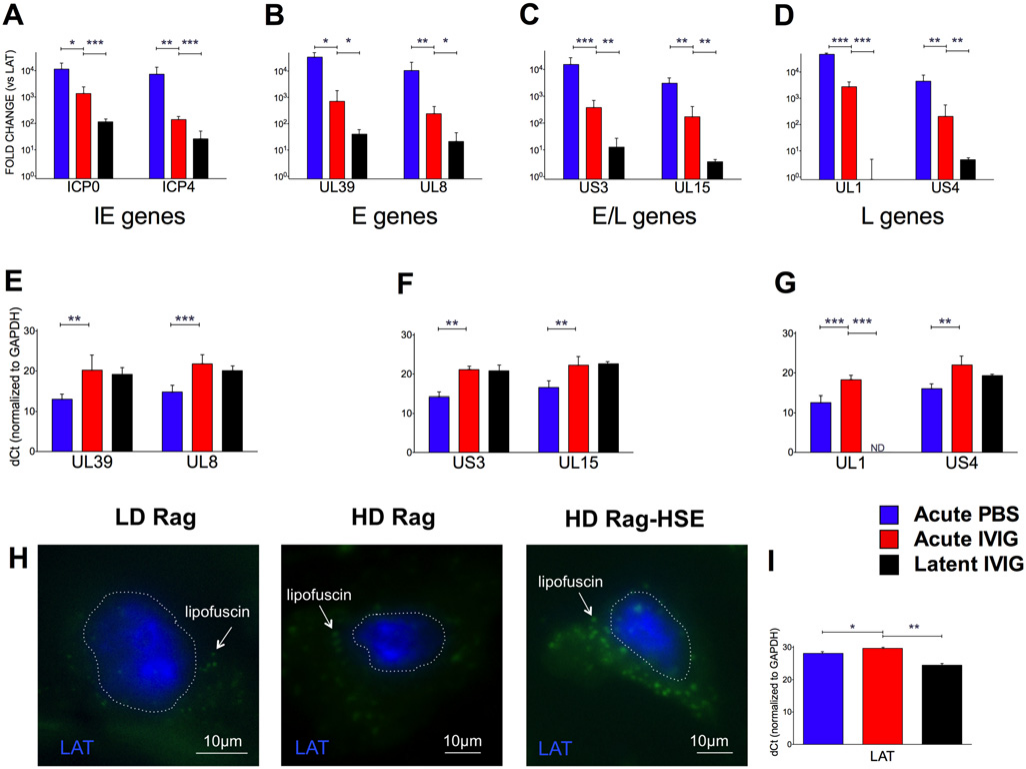
Acute and latent gene expression in HD infected B6-Rag trigeminal ganglia. qRT-PCR analysis of RNA from Tg collected from acute (day 5) PBS (blue bars) or IVIG treated (red) and latent (day 60) IVIG treated (black) HD B6-Rag mice are shown as fold-change relative to LAT expression (A-D) for Immediate Early (A), Early (B), Early/Late (C) and Late (D) genes, and delta-Ct (dCt) values normalized to GAPDH expression for Early, Early/Late and Late genes (E, F, and G, respectively). Tg sections obtained from latently infected LD, HD, and a HD Rag mouse showing signs of HSE were processed for RNA-FISH using a LAT RNA probe **(H)**. The blue signal stains for LAT, the green signal comes from neuronal cytoplasmic lipofuscin (aggregates) autofluorescence. The dotted lines outline the nuclei. Wide-field imaging. Scale bar = 10 μm. **(I)** GAPDH normalized dCt values for LAT expression in HD Rag mice determined using RT-PCR (I). Statistical analysis is described in methods.

To confirm establishment of latency in both LD and HD B6-Rag mice, we performed FISH to visualize LAT transcripts in the nuclei of sensory neurons from latently infected healthy mice, as well as in a HD mouse that spontaneously reactivated and presented symptoms of HSE (Fig. 5H). The transition to latency in HD Rag mice was characterized by a marked decrease in the ratio of lytic to LAT transcripts for HSV1 genes representative of Immediate Early (IE), Early (E) Early/Late (E/L) and Late (L) kinetic classes of genes (Fig. 5A-D). Comparing HD PBS to HD IVIG at day 5 pi (acute infection) shows that IVIG inhibited lytic gene expression significantly (Fig. 5E-G) but interestingly had less of an effect on LAT expression (Fig. 5I). IVIG given at 24 h pi effectively abolished acute stage HSV1 replication (day 5) in the Tgs of HD and LD Rag mice (Table 1 and S1 Fig.). Also, infectious virus was undetectable in Tgs and BS of latently infected HD Rag mice (during and after cyclic IVIG treatment) that were randomly euthanized at different times (Fig. 3A). Consistent with these findings, acute and latent stage levels of selected lytic transcripts were not different for IVIG treated HD Rag mice (Fig. 5E-G). The ratio of lytic to LAT transcripts was similarly decreased for LD Rag mice during transition to latency, though robust data was obtained for fewer genes (S3A Fig). Importantly, LAT levels increased significantly by ~28 and 37 fold during the transition to latency for both LD and HD B6-Rag mice respectively (S3C Fig), resulting in similar levels of LAT accumulation during latency in LD and HD Rag mice (Figs. 5I and S3B).

### Utility of the Model: Memory T Cells Are Required to Control Reactivation

To confirm the widely accepted view that T cells are required to control HSV1 reactivation, we transferred total spleen cells or the CD3^+^ T cell fraction from HSV1 immunized or non-immunized wt mice into latently infected HD inoculated 129-Rag and B6-Rag mice. Latently infected Rag mice were transferred with spleen cells or the CD3^+^ T cell fraction and 72 h later we induced IVR and monitored the mice for signs of HSE. All B6-Rag and 129-Rag mice transferred with total spleen cells or the CD3^+^ T cell fraction obtained from HSV1 immunized mice survived (Fig. 4E and 4F). Latent HD inoculated Rag mice transferred with naïve total spleen cells and non-transferred control latently infected Rag mice all succumbed (Fig. 4E and 4F). Interferon-γ (IFN-γ) is not essential for T cell mediated inhibition of HSV1 replication as both latent B6 wt and GKO mice survived following IVR (S4 Fig) [14]. Similarly, we reported previously that although HSV1 reactivation was augmented in GKO mice on the susceptible 129-background all the mice nonetheless survived [14], which indicates the involvement of redundant mechanisms in control of reactivated HSV1. Importantly, only HSV1 specific memory T cells were effective in preventing reactivation as the CD3^-^ spleen cell fraction, comprised predominantly of B cells including HSV1 memory B cells, was not protective (Fig. 4E).

## Discussion

We exploited the established immunomodulatory and antiviral activities of IVIG to establish a new model of latent HSV1 infection in immunodeficient Rag mice. Though not central to our studies, it was necessary to examine aspects of the mechanism(s) of IVIG protection of Rag mice to derive the model. To set up the model, we treated HSV1 infected 129-Rag and B6-Rag mice with IVIG to promote long-term survival with latent infections. A key finding was that IVIG protection was strictly dependent on the HSV1 inoculum dose. Thus, we could define a threshold HSV1 inoculum, referred to as LD, above which a single dose of IVIG given 24 h pi failed to promote long-term survival of latently infected Rag mice. Analysis of viral Tg titers and BS inflammatory infiltrates revealed that for LD infections, IVIG protected 129-Rag and B6-Rag mice primarily by suppressing HSV1 replication as CNS inflammation was respectively mild or essentially absent [22,27].

Despite the HD inoculum being only 3-fold higher than the LD inoculum, suppression of virus replication and CNS inflammation resulting in long-term survival of Rag mice with latency required 3 more doses of IVIG after the initial dose at 24 h pi and, to ensure complete eradication of infectious HSV1, a 10-day course of ACV treatment was also given. Increased CNS inflammation was also seen in the B6-Rag mice inoculated at HD, though of lower magnitude than in 129-Rag HD mice. Random screening of healthy latently infected HD B6-Rag mice was routinely negative for infectious HSV1 in the Tg and BS confirming latency, hence we attribute the occasional sporadic death of mice to spontaneous reactivation since significant virus loads were detected in the Tg and BS of mice that died. Importantly, the production of lytic transcripts in a majority of latently infected neurons isolated by laser capture from non-HS treated wt mice was recently reported [31,32]. In a related study [33], spontaneous reactivation resulting in production of very low levels of infectious HSV1 by very few neurons in the Tg has been observed to occur frequently in latently infected wt mice and in Rag mice this would be expected to result in unimpeded virus replication and eventually death, consistent with our results for HD Rag mice. Similar to our results, three doses of a neutralizing mAb targeting a gB epitope given starting 24 h pi protected most HSV1 F strain infected NOD/SCID mice long-term [34]. Since the HSV1 F strain does not induce exaggerated CNS inflammation, we infer protection depends on inhibition of HSV1 replication and indeed this mAb suppressed plaque formation in monolayer cultures [34]. Although IVIG usually has significant neutralizing antibody titers, we infer that during cyclic treatment IVIG suppresses intracellular HSV1 replication by an unknown mechanism(s) because HSV1 spreads exclusively via axonal transport in the PNS and CNS [35]. This is not without precedent as IVIG has been reported to inhibit HSV1 and HCMV plaque formation in monolayer cultures when added after virus is internalized [36,37]. Additionally, a pool of non-neutralizing monoclonal antibodies was shown to suppress growth of HSV1 in acutely infected Tgs cultured *in vitro* [38].

We defined a HD inoculum threshold that is 3-fold higher than the LD inoculum and also 3-fold higher for B6-Rag compared to 129-Rag mice. Cyclic IVIG treatment of HD mice facilitated establishment of latent infections that were readily reactivated *in vivo* by HS resulting in the majority of 129-Rag and B6-Rag mice succumbing to HSE. Importantly, despite efficient *in vivo* HSV1 reactivation in all Tgs of LD mice resulting in transient production of infectious virus, all the mice survived and infectious HSV1 was undetectable in Tgs assayed day 21 after HS. BS were not assayed but in assaying necropsy Tg and BS from mice that succumbed to spontaneous reactivation we observed instances where infectious virus was present only in the BS (Fig. 2A), which is consistent with reactivation also occurring in the BS as reported recently for mice subjected to HS [30]. Overall, the data show HSV1 was efficiently reactivated *in vivo* in both LD and HD latently infected Rag mice, but remarkably was amplified resulting in uncontrolled replication and fatal HSE only in HD but not LD Rag mice.

Although the propensity to reactivate, measured by transient production of infectious HSV1 in the Tgs of wt mice, has been correlated with latent HSV1 Tg genomic loads [39–41], differences in the latent genomic load cannot explain why fatal HSE developed after IVR in HD but not LD Rag mice, because HSV1 was reactivated *in vivo* in both groups of mice. The establishment of latency in LD and HD B6-Rag mice showed characteristic changes in lytic and LAT gene expression similar to what has been reported for immunocompetent wt mice transitioning from acute to latent infection [2,42]. Thus, LAT levels were significantly elevated concomitant with a reduction in the ratio of lytic to LAT gene transcript levels, particularly for HD mice. Most importantly, changes in the ratio of lytic to LAT transcripts were similar for LD and HD Rag mice transitioning to latent stage infection and as accumulated latent LAT levels were indistinguishable, it appears latency establishment was qualitatively the same for the LD and HD Rag mice.

It is increasingly evident that neurons express intrinsic restriction factors to protect themselves from virus infections and differences in these factors among neuronal subtypes confers differential susceptibility to virus infection [35,43–49]. Thus, impaired intrinsic TRL3 and UNC-93B-dependent IFN-α/β antiviral responses in neurons and oligodendrocytes were identified as critical factors predisposing children to development of HSE [50]. Since, HS induced production of low levels of infectious HSV1 in latently infected Tgs in LD Rag mice that all survived, we infer that virus amplification and spread were rapidly suppressed by intrinsic neuronal restriction mechanism(s) thus precluding development of HSE. Strong support for this contention comes from the recent report of intrinsic neuronal restriction mechanisms suppressing HSV1 replication [44], thereby facilitating latency establishment in neurons of human dorsal root ganglia infected *ex vivo* with HSV1 and subsequently maintained as long term xenografts in SCID mice lacking B and T cells [28]. In HD Rag mice, the presumption is that the intrinsic neuronal restriction mechanisms are overwhelmed or compromised by mechanism(s) yet to be determined resulting in virus amplification and spread, culminating in death of the majority of mice. In this regard, it is interesting that infiltrating inflammatory cells accumulated in BS of HD but not LD mice that supported only transient virus production after IVR. The neuronal intrinsic restriction mechanisms are likely more effective in B6-Rag compared to 129-Rag mice based on differences in (i) the respective LD_50_, (ii) both the LD threshold inoculum for protection by a single dose of IVIG and (iii) the HD threshold inoculum that supports efficient IVR resulting in fatal HSE being roughly 3-fold higher for B6-Rag than 129-Rag mice.

Our adoptive transfer results during cyclic IVIG treatment are consistent with T cells being primarily required for clearing all traces of infectious HSV1 during acute infection and suppressing spontaneous reactivation as previously proposed [51,52]. Interestingly, CD8+ T cell control of reactivated virus was impaired in mice infected with recombinant HSV1 KOS strains expressing either the HCMV US 11 or MCMV m152 genes that effectively inhibited MHC I surface expression compared to HSV1 ICP47[53]. Since the functional HSV1 homologue ICP47 is effective in inhibiting MHC I expression in human but not murine cells, this may account for the lower spontaneous reactivation rates observed for HSV1 in murine models relative to humans [54]. An important point emerging from our studies is that while T cell control is crucial for eliminating spontaneously reactivated virus in HD infections it is dispensable for maintaining latency in LD infections, particularly for resistant B6 mice as we previously reported 50% survival for immunodeficient B6 mice with latent infections independent of treatment with ACV or IVIG [22]. Relevant to this study, is that although 1 of 5 mice controlled virus by day 7 pi this mouse succumbed to spontaneous reactivation on day 77. Though the other 4 mice shed virus in the tear film up to 14 day pi, three mice eventually controlled the virus and survived for 11 weeks (Table 2 in [22]) and latency was confirmed by Tg explant reactivation in 2 mice sacrificed on day 80 pi. These prior data support the existence of potent neuronal intrinsic restriction mechanisms and show that neither T nor B cells are mandatory for establishment and maintenance of latency, consistent with results presented here and studies with SCID mice [28,42].

The widely accepted view that CD8^+^ memory T (Tm) cells residing locally in latently infected sensory ganglia are critical for suppressing HSV1 reactivation, acting by non-lytic and lytic mechanisms that include secretion of IFN-γ, perforin and granzyme B [17,55–57] is supported mainly by circumstantial evidence. We provided direct evidence that HSV1 Tm but not naive CD3^+^ T cells or memory B cells adoptively transferred 72 h prior to HS induction of IVR are critical for inhibiting HSV1 reactivation in latently infected heat stressed HD Rag mice. The Tg CD8^+^ tissue resident memory (Trm) T cell pool recovered independently of recruitment from peripheral HSV1 specific CD8^+^ T cells in the blood when disrupted [58]. Notably, adoptive transfer of CD8^+^ Tm suggested that once established this pool might be inaccessible to circulating CD8^+^ Tm or T effector cells [58,59]. Our adoptive transfer results raise the intriguing possibility that the Tg Trm CD8^+^ T cells might contribute to restricting access of circulating Tm to latently infected Tgs in wt mice [60]. In our model, HS is acting independently of T cells to induce IVR and it might be that by stressing neurons, it triggers proapoptotic signals resulting in global derepression of HSV1 gene expression and rapid induction of virus replication [61]. This possibility could be addressed by studying reactivation using an appropriate lineage tracing strain that facilitates genetic tagging of latently infected neurons on the Rag^-/-^ background.

Overall, the B6-Rag latency model is preferred to the 129-Rag model because latency is more stable; hence fewer mice are lost due to spontaneous reactivation during establishment of latency. Additionally, most knockout mice for immunologically important genes are available on the B6 but not the 129 genetic background and these mice will be an important resource for leveraging the power of this latency model to address numerous questions relating to immune control of HSV1 latency.

**Fig 6:**
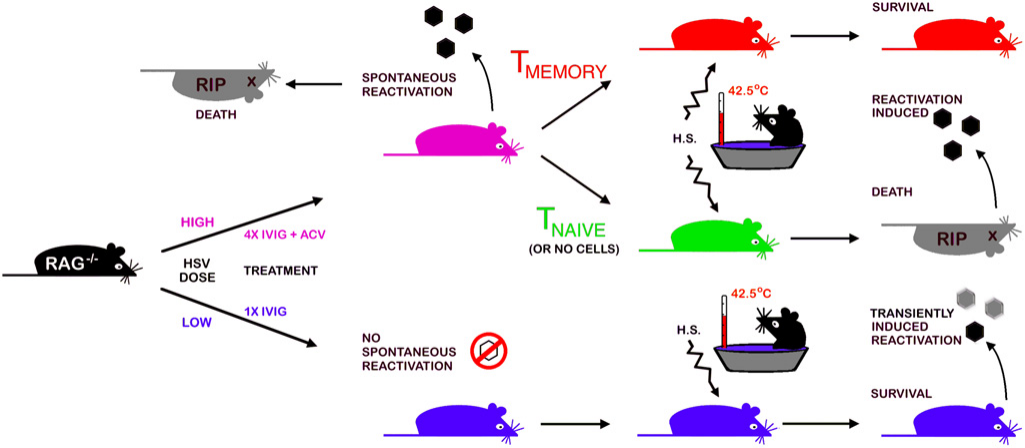
A schematic depicting the Rag-latency model. Details of establishment and characterization of latency in B6-Rag mice, in vivo reactivation and adoptive transfer of T cells into latently infected Rag mice are presented in the text and methods.

The strengths of the Rag latency model illustrated schematically in Fig. 6 include (i) the robust *in vivo* reactivation phenotype, namely development of HSE at a high rate that can be exploited in screens to identify new drugs to block reactivation, (ii) the potential to study HSV genes implicated in controlling establishment of or reactivation from latency, (iii) the ability to modify the model by judicious crosses with selected knockout strains to validate *in vivo* the role of specific viral and cellular genes that have been implicated in control of latency in *in vitro* latency models. We used the virulent HSV1 17+ strain to set up the model as we used this strain in prior studies of IVIG protection against HSE but, using a less virulent strain may increase the efficiency of latency establishment in Rag mice treated with IVIG without compromising efficient *in vivo* reactivation resulting in disease symptoms but possibly with reduced mortality.

## Materials and Methods

### Ethics Statement

This study was carried out in strict accordance with the recommendations in the *Guide for the Care and Use of Laboratory Animals* of the National Institutes of Health. All animal studies were conducted under a protocol approved by the Institutional Animal Care and Use Committee (IACUC, Permit # A3001–01) of City of Hope to ensure the highest ethical and humane standards were followed.

### Mice and HSV1 Infection

Mice used in this manuscript include the 129-Rag2^-/-^ (129S6/SvEvTac-Rag2tm1Fwa; #RAG2), B6-Rag2^-/-^ (B6.129S6-Rag2tm1Fwa N12; #RAGN12) and 129S6 (129S6/SvEvTac, #129SVE) that were obtained from Taconic Farms Inc. (NY, USA). Derivation of IFN-γ^-/-^ (Gko) mice on the 129-SvEv background has been described previously [62]. OT-I, (C57BL/6-Tg(TcraTcrb)1100Mjb/J, #003831) which expresses a transgenic T cell receptor (Tcra-V2, Tcrb-V5) that recognizes OVA_257–264_ in the context of H-2K^b^ and C57BL/6J (#000664) were obtained from Jackson Laboratory (JAX-West) USA.

6–10 week old mice were inoculated with the indicated doses of HSV1 17+ strain by corneal scarification as previously described [22]. Virus titers in tissue homogenates were determined by plaque assay on Vero (African Green monkey) cell monolayers. Virus release assay was performed to detect infectious virus in the Tg at different times pi. Briefly, virus in Tgs homogenates was detected on Vero cells without addition of human sera to limit secondary viral spread. If no virus was detected in the first round of infection, cells were frozen and thawed three times. Virus was detected in the supernate on Vero cells using a second round of infection. The City of Hope IACUC approved of all animal procedures.

**Low dose and high dose HSV1 infection of rag mice.** The LD_50_ for 129-Rag and B6-Rag mice is 30 and 100 PFU respectively. LD Rag mice refer to mice inoculated at ≤32x LD_50_ that were injected ip with 4mg IVIG (Carimmune, CSL Behring, PA) 24 h pi. HD Rag mice refers to mice inoculated at 100x LD_50_ that were given IVIG 24 h pi then three additional doses of IVIG given every 12 days supplemented with a 10-day course of daily acyclovir (ACV, 50mg/kg in PBS) ip injections beginning on day 10 pi [22].

### Assessing CNS Inflammatory Responses and Adoptive Transfers

Mononuclear cells were isolated from 2–3 pooled BS as previously described [27]. Surface marker expression on BS CD45^high^ infiltrating cells from HSV1 infected mice was determined by flow cytometry. CD45^high^ low side scatter (SSC) CD11b^+^ Ly6C^high^ CD107a^+^ monocytes were considered as inflammatory monocytes. In some experiments, HD Rag mice received CD3^+^ T cells or CD3^-^ cells isolated from spleens of naïve or HSV1 immunized 129 or B6 wt mice or from H-2K^b^ restricted OVA_257–264_ specific B6 OT-I CD8^+^ TcR transgenic mice, purified using kits (Stemcell Technologies, Vancouver, Canada). HSV1 specific CD8 T cells composed 5–8% of the total CD8 fraction in the spleens of immunized mice as detected by reactivity to the immunodominant H2-K^b^-restricted HSV1 gB_498–505_ tetramer [63].

### Hyperthermia Stress to Induce In Vivo Reactivation

Latently infected 129 and Gko mice were derived by infecting mice at 3200 PFU with HSV1–17+ and injecting them with 4 mg IVIG at 24 h pi. At day 25 or later after establishment of latency, 129 wt, Gko, LD and HD latent B6- and 129-Rag mice were subjected to hyperthermic stress in a water bath (42.5°C) essentially according to the protocol of Sawtell and Thompson [15] as described previously [14]. Mice were monitored twice a day for signs of HSE and euthanized as necessary.

### qRT-PCR Analysis of HSV1 Gene Expression in Acutely and Latently Infected Tgs

Total RNA was isolated from the ipsilateral Tgs using TRIzol (Invitrogen, Carlsbad, CA). Briefly, tissues were homogenized in TRIzol solution using a Bio-Gen PRO200 homogenizer (PRO Scientific, Oxford CT) before addition of chloroform. Samples were centrifuged at 4°C for 15 min at 14,000 rpm and the RNA-containing layer was aspirated and incubated with 100% isopropanol for 10 min at room temperature. The Qiagen RNeasy Mini Kit (Qiagen, Valencia, CA) was used to further purify the RNA at this stage and samples were treated twice with DNAse to eliminate contaminating DNA. RNA samples were resuspended in 40 uL nuclease free water and their concentration determined using a NanoDrop 1000 spectrophotometer (NanoDrop Products, Wilmington, DE). After RNA isolation, 150 ng of total RNA was reverse-transcribed using both Oligo (dT) primers and random hexamers. Quantitative RT-PCR for selected HSV genes was performed using 2X SYBR Green Mix (Life Technologies, Grand Island, NY) in a standard two-step (30s at 67°C for ICP0 and ICP4, 58°C for GAPDH, 60°C for UL1 and UL39 and 62°C for the other genes, and 15s at 94°C) PCR, and delta Ct values were determined by normalizing transcript expression to the housekeeping gene GAPDH. LAT amplification was done using a probe PCR (2-step PCR of 95°C for 15s/56°C for 60s). S1 Table shows primer sequences used. Statistical analysis for pairwise comparisons of Acute PBS vs Acute IVIG and Acute IVIG vs Latent IVIG was done using GAPDH normalized Ct values adjusted for LAT where appropriate as unpaired t-tests with Welch's correction for unequal variances (* p<0.05, ** p<0.01, *** p<0.001).

### RNA-FISH for Visualization of LAT Expression in Tg Sections

Mice were anesthetized at appropriate times after infection procedure, then individual Tg were collected and snap-frozen before embedding in OCT. 10 μm sections were collected on glass slides, and stored at -80°C. RNA-FISH probe labeling and RNA-FISH procedures were performed as described previously [64,65]. Briefly, biotinylated single-strand LAT RNA probe was prepared by *in vitro* transcription using plasmid pSLAT-2 [66]. Frozen sections were thawed and treated using solutions containing 2 mM of the RNAse inhibitor ribonucleoside vanadyl complex, i.e., rehydrated in 1X PBS, fixed with 2% paraformaldehyde, and permeabilized in 0.5% Triton X-100. Sections were then dehydrated by successive incubations in 70% to 100% ethanol baths, before drying and overnight hybridization at 65°C with 60 ng of LAT RNA probe in a 50% formamide buffer. After several washes in 2 X SSC buffer, detection was performed by incubation of the samples with HRP-streptavidin, followed by TSA amplification (Invitrogen) with an AlexaFluor 350 conjugated substrate. All sections were mounted under coverslip using Vectashield mounting medium (Vector Laboratories) and stored at +4°C until observation.

## Supporting Information

S1 Fig IVIG suppresses virus replication in LD B6-Rag mice. LD infected B6-Rag mice treated with a single dose of 4 mg IVIG or PBS at 24 h pi were euthanized at different times pi for determination of virus titers in the right Tg (n = 5 / group).
(TIF)

S2 Fig HSV1 genomic DNA copy number in latently infected ganglia in Rag mice. Genomic HSV1 DNA copy numbers for DNA from latently infected Tgs from 129-Rag (circles) or B6-Rag (squares) mice are shown after LD (open symbols) or HD (filled symbols) infections as described in Supplemental Materials and Methods. Inter-strain differences were evaluated by Student’s t-test. Data is representative of two experiments with n = 3–5 mice per group.
(TIF)

S3 Fig Acute and latent gene expression in LD B6-Rag trigeminal ganglia. qRT-PCR analysis of RNA from Tg collected from acute (day 5) PBS (blue bars) or IVIG treated (red) and latent (day 28) IVIG treated (black) LD B6-Rag mice are shown as fold-change relative to LAT expression for selected acute genes (A), LAT expression normalized to GAPDH expression (B) and a side-by-side comparison of fold increase of LAT expression during latency relative to acute (day 5) LAT expression for LD and HD B6-Rag mice (C).
(TIF)

S4 Fig IFNγ is dispensable for T cells to control reactivated HSV1. 129 WT and IFNγ^-/-^ mice were infected with 3200 PFU of HSV 17+ strain and given 4 mg IVIG at 24 h pi. At day 60 pi, virus was reactivated in all surviving mice by HS and survival was monitored (n = 10–14).
(TIFF)

S1 Table Primer sequences used for SYBR Green and probe PCR. (DOCX)

S1 Text Supplemental materials and methods. (DOCX)
