# Supplementary figures and images for: Establishment of HSV1 Latency in Immunodeficient Mice Facilitates Efficient In Vivo Reactivation
Source: PLoS Pathog. 2015 Mar 11;11(3):e1004730. doi: 10.1371/journal.ppat.1004730 (PMC4356590; doi:10.1371/journal.ppat.1004730)

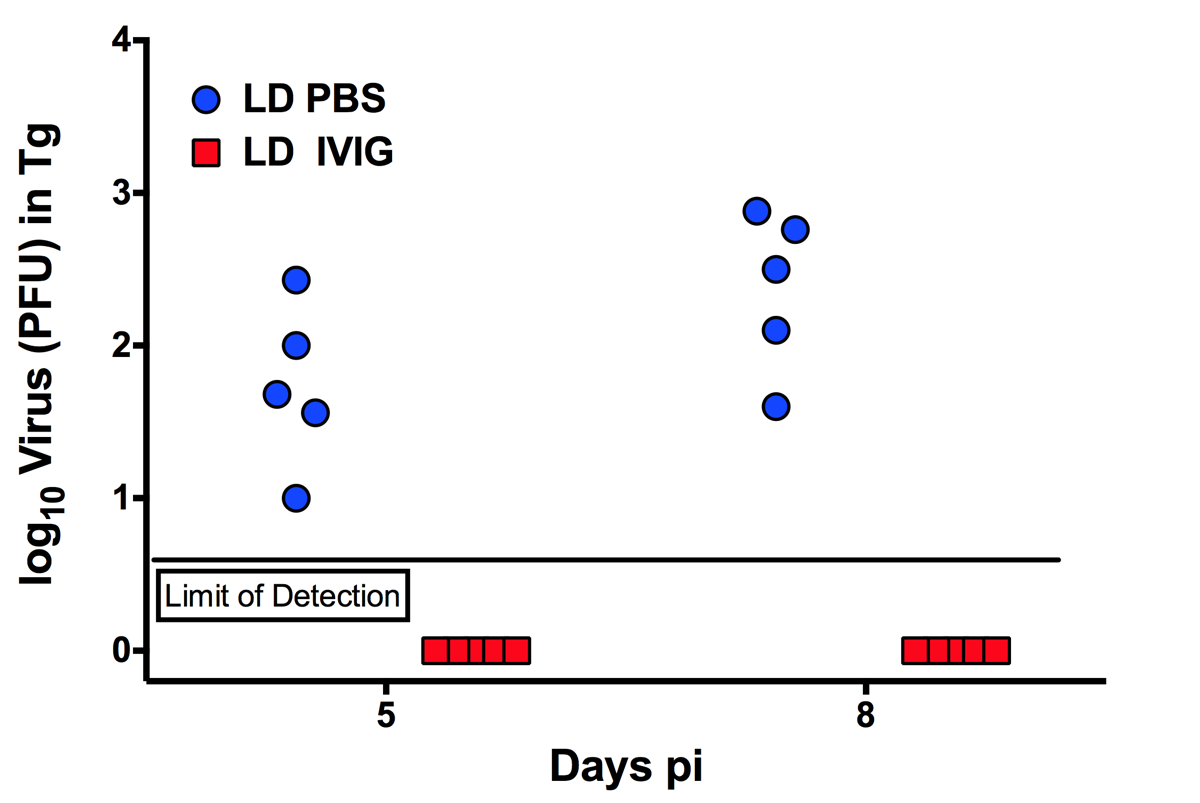

Supplement: S1 Fig — LD infected B6-Rag mice treated with a single dose of 4 mg IVIG or PBS at 24 h pi were euthanized at different times pi for determination of virus titers in the right Tg (n = 5 / group). (TIF) [file ppat.1004730.s001.tif]

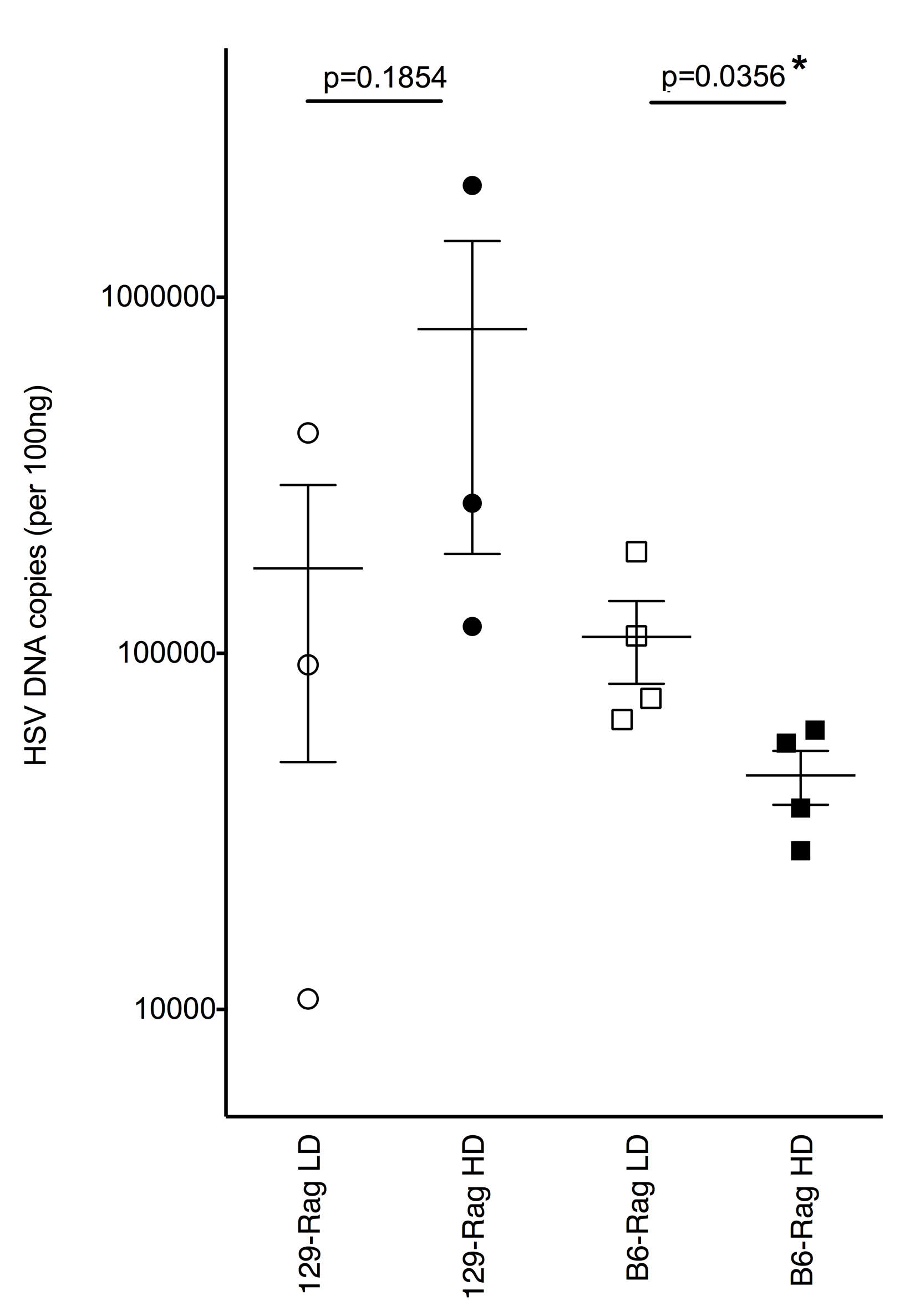

Supplement: S2 Fig — Genomic HSV1 DNA copy numbers for DNA from latently infected Tgs from 129-Rag (circles) or B6-Rag (squares) mice are shown after LD (open symbols) or HD (filled symbols) infections as described in Supplemental Materials and Methods. Inter-strain differences were evaluated by Student’s t-test. Data is representative of two experiments with n = 3–5 mice per group. (TIF) [file ppat.1004730.s002.tif]

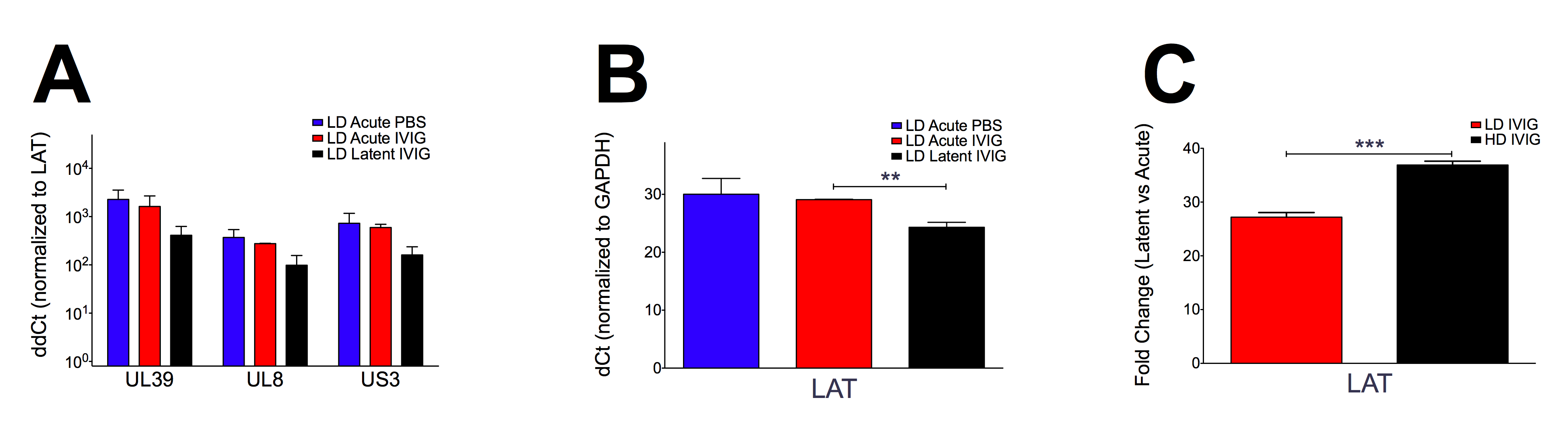

Supplement: S3 Fig — qRT-PCR analysis of RNA from Tg collected from acute (day 5) PBS (blue bars) or IVIG treated (red) and latent (day 28) IVIG treated (black) LD B6-Rag mice are shown as fold-change relative to LAT expression for selected acute genes (A), LAT expression normalized to GAPDH expression (B) and a side-by-side comparison of fold increase of LAT expression during latency relative to acute (day 5) LAT expression for LD and HD B6-Rag mice (C). (TIF) [file ppat.1004730.s003.tif]

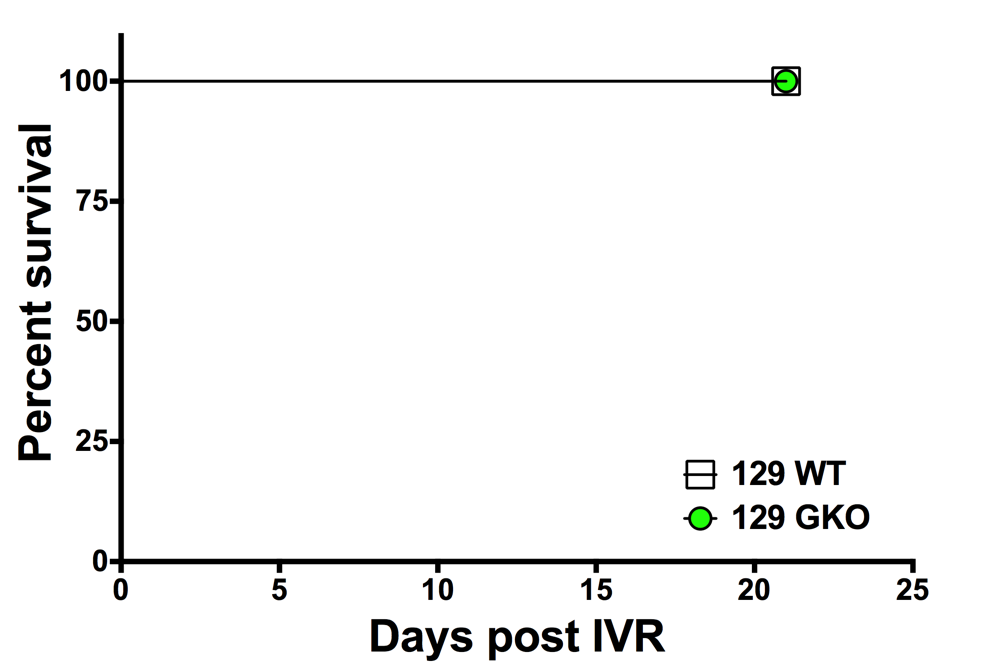

Supplement: S4 Fig — 129 WT and IFNγ-/- mice were infected with 3200 PFU of HSV 17+ strain and given 4 mg IVIG at 24 h pi. At day 60 pi, virus was reactivated in all surviving mice by HS and survival was monitored (n = 10–14). (TIFF) [file ppat.1004730.s004.tiff]
