# Supplementary material for: Establishment of HSV1 Latency in Immunodeficient Mice Facilitates Efficient In Vivo Reactivation
Source: PLoS Pathog. 2015 Mar 11;11(3):e1004730. doi: 10.1371/journal.ppat.1004730 (PMC4356590; doi:10.1371/journal.ppat.1004730)
Supplement: S1 Table — (DOCX) [file ppat.1004730.s005.docx]

| **Supplemental Table 1. Primer sequences used for SYBR Green and probe PCR.** | | | |
| --- | --- | --- | --- |
| **Gene** | **Direction** | **Sequence (5' to 3')** | **Annealing Temperature** |
| ICP0 | F | GCGGACAGCACGGACACGGAACTG | 66.2 C |
| ICP0 | R | CGCTGATTGCCCGTCCAGATAAAGTCCA |  |
| ICP4 | F | GGCGGCGGCGACGACGACGATAA | 66.6 C |
| ICP4 | R | GAGTACAGCACCACCACGCGCACGTCCT |  |
| UL1 | F | CGCTCCCGTCTGACGATCTTG | 60.2 C |
| UL1 | R | GTCCGACGTGGCGATGATG |  |
| UL8 | F | CCCCGGTAAACACCAACTCAATCTC | 62.3 C |
| UL8 | R | GCCTGCGACCGCCTTATCTT |  |
| UL15 | F | CAC CAA CAC CGG GAA GGC CAG TAC G | 63.2 C |
| UL15 | R | AAA GTG CTC CAG GGC GAA GAT GAT AT |  |
| UL39 | F | GTGAACCGCCACTACATCTACGACA | 60.8 C |
| UL39 | R | GCCGCCCAAAGTCAAACGTCT |  |
| US3 | F | CCTTGCCCAGATGTACGGAAAC | 61.6 C |
| US3 | R | CCGGCTGCGTCGTCGTAT |  |
| US4 | F | CCGCCATCAGCCTTACCAC | 61.8 C |
| US4 | R | GTATGTTGAGGCGTCGGAACCA |  |
| GAPDH | F | GGTGAGGCCGGTGCTGAGTATGTC | 61.7 C |
| GAPDH | R | CATGAGCCCTTCCACAATGCCAAAG |  |
| LAT | F | GGG TGG GCT CGT GTT ACA G | 56.9 C |
| LAT | R | GGA CGG GTA AGT AAC AGA GTC TCT A |  |
| LAT | Probe | 6FAM-ACA CCA GCC CGT TCT TT-BHQ1 | 54.4 C |
|  | | | |
