# Supplementary material for: Establishment of HSV1 Latency in Immunodeficient Mice Facilitates Efficient In Vivo Reactivation
Source: PLoS Pathog. 2015 Mar 11;11(3):e1004730. doi: 10.1371/journal.ppat.1004730 (PMC4356590; doi:10.1371/journal.ppat.1004730)
Supplement: S1 Text — (DOCX) [file ppat.1004730.s006.docx]

**Establishment of HSV1 Latency in Immunodeficient Mice Facilitates Efficient *In Vivo* Reactivation**

Chandran Ramakrishna, Thanh K. Nguyen, Patric S. Lundberg Harry Openshaw and *Edouard M. Cantin

**Supplemental Materials and Methods:**

**Quantifying Genomic Load in the Trigeminal ganglia (Tg)**

Quantitative PCR for gG was performed as previously described [Lundberg, 2003 #4805]. Briefly, Tgs harvested from n = 3 - 5 latently infected LD and HD Rag mice at day 25 pi were homogenized and total ganglionic DNA was extracted from Tg homogenate pellets by digestion of the tissue with proteinase K followed by phenol extraction and ethanol precipitation and DNA concentration and purity were determined using a NanoDrop spectrophotometer (Thermo, Wilmington, DE). The TaqMan-based PCR assay conditions for mouse Tg DNA included the use of 4 mM Mg2+, 500 nM primers, 5% dimethyl sulfoxide, 2 U of AmpliTaq Gold, TaqMan probe at 100 nM and using 100 ng of total ganglionic DNA per reaction. The gG-specific primers and TaqMan probe sequences were: 5’-CTGTTCTCGTTCCTCACTGCCT-3’, 5’-CACAAAAACGATAAGGTGTGGATG-3’, and 5’-FAM-CCC TGG ACA CCC TCT TCG TCG TCA G-BHQ1-3’ (IDT, Coralville, IA). Real-time PCR was run on the BioRad CFX96 Real Time System (BioRad, Hercules, CA) using a two-step PCR (94/6^o^C) for 40 cycles and for each amplification, a standard curve, generated by amplification of purified HSV1 DNA ranging from 5 to 5*107 copies in 100 ng of normal mouse DNA, was linear throughout the range (r2>0.995). All standards were in performed triplicate, all samples were in duplicate, and PCR standard inter-assay correlation coefficient variability was less than 1.2%.
